# Bioconversion of biowaste by black soldier fly larvae ( *Hermetia illucens* L.) for dried larvae production: A life cycle assessment and environmental impact analysis

**DOI:** 10.12688/f1000research.132371.1

**Published:** 2023-07-11

**Authors:** Rudy Agung Nugroho, Muhammad Nasir Rofiq, Arif Dwi Santoso, Ahmad Ismed Yanuar, Rahmania Hanifa, Nadirah Nadirah

**Affiliations:** 1 Animal Physiology, Development, and Molecular Laboratory, Department of Biology, Faculty of Mathematics and Natural Sciences, Mulawarman University, Samarinda, Kalimantan TImur, 75123, Indonesia; 2 Research Center for Sustainable Production System and Life Cycle Assessment, The National Research, and Innovation Agency (BRIN), Tangerang Selatan, Banten, Indonesia; 3 Environmental Engineering Study Program, Department of Civil and Environmental Engineering, Faculty of Engineering, Universitas Indonesia, Depok, West Java, Indonesia

**Keywords:** Bioconversion, Hermetia illucens, LCA, Sustainable

## Abstract

**Background**: *Hermetia illucens* L. have gained popularity in recent years as an environmentally friendly response to both the present and potential future food/feed crisis. The larvae of *H. illucens* L., or black soldier fly larvae (BSFL), is an alternative solution to tackle the issue of organic waste bioconversion. However, understanding the environmental loads associated with biowaste bioconversion using BSFL to produce dried BSFL is a pivotal point to keep the environment sustainable. This study reported a life cycle assessment (LCA) of the biowaste bioconversion process of BSFL and determined the environment impact analysis to make recommendations for modifications to lessen environmental consequences.

**Methods**: The methodology used is life cycle assessment (LCA), which includes: (a) system boundary determination (gate-to-gate), starting from biowaste production, biowaste bioconversion, prepupae and BSFL frass production. The system boundary of the dried BSFL production is designed for both the processing and production of one cycle of BSFL; (b) life cycle inventory activities carried out at PT Biomagg Sinergi Internasional, Depok, West Java, Indonesia; (c) conducting life cycle impact assessment on five environmental impact categories namely global warming potential (GWP), acidification (AC), terrestrial eutrophication (TE), fossil fuel depletion (FFE), eco-toxicity (ET); and (d) interpretation of the assessment result. The LCA is conducted using openLCA 1.11 software and TRACI 2.1 impact assessment method.

**Results**: The impact values of GWP, AC, TE, FFE, and ET, per 100 kg of BSFL dried production was 6.687 kg CO _2_ eq; 0.029 kg SO _2_-eq; 0.092 kg N-eq; 16.732 MJ surplus; 121.231 CTUe. Production of prepupa had the highest hotspots in these emissions, followed dried BSFL production.

**Conclusions:** Efforts to reduce environmental impacts that can be done are by implementing an integrated rearing system using substrate from a single type of known substrate for BSFL and using alternative drying methods for BSFL dried production.

## Introduction

The popularity of farmed insects as a future source of food, feed, and energy is growing. ^1^^–^^3^ Insects have been identified as a viable answer to the worldwide difficulties connected with a shortage of protein sources for feed and food as the world population grows. ^4^ In recent years, there has been considerable growth in the number of research and commercial advances related to the use of insect production in connection to recycling, reduce, and reuse of agri-food system side-streams and waste biomass. ^5^^–^^7^ Insects have a higher feed conversion efficiency and fewer greenhouse gas emissions than traditional cattle, as well as a nutritional content that makes them potentially acceptable for food and animal feed. ^8^

Further, the United Nations Food and Agriculture Organization has recognized the potential of edible insects to contribute to healthy and sustainable diets and has urged their inclusion in the diets of people all over the globe. ^9^ The black soldier fly (BSF) or *Hermetia illucens* is a focus species among farmed insects owing to the ability of its larvae (BSFL) to rapidly thrive on various organic waste streams. ^10^^,^^11^ The BSFL consumes a vast amount of organic waste and converts it into larval biomass, which may later be converted into animal feeds. ^12^ As commonly employed bio-converter agents for diverse organic waste, the BSFL are often used as feed for poultry and fish because of their high protein content. ^13^ The protein content of the BSFL range from 40–44%, and is rich in amino acid, which is better compared to soybean meal. ^14^ Besides protein and amino acid, dietary BSFL oil is beneficial to enhance feed conversion ratio and increase the incorporation of medium-chain fatty acids into abdominal fat pad and serum antioxidant capacity specifically in broiler chickens. ^15^

Despite several literature sources on economic feasibility and societal acceptability, many unanswered topics remain for academics to investigate. Industrial activities (for example: PT Biomagg, Sinergi Internasional, a BSF farm located in Depok, West Java, Indonesia) will certainly have an impact on the environment, such as changes in the quality of water, soil, and air. To reduce pollution and environmental impacts that occur during the product life cycle, the appropriate method for analyzing is a life cycle assessment (LCA). LCA analysis aims to calculate the environmental load based on an inventory analysis of the use of resources, energy, air, fuel, and others so that the environmental burden can be identified and then analyzed using different alternatives to reduce the impact. ^16^^–^^18^ The present study reported to identify and analyze input output based on inventory data from BSFL dried products and determine potential environmental impacts in the form of global warming potential (GWP), acidification (AC), terrestrial eutrophication (TE), fossil fuel depletion (FFE), and ecotoxicity (ET).

## Methods

**Figure 1.**
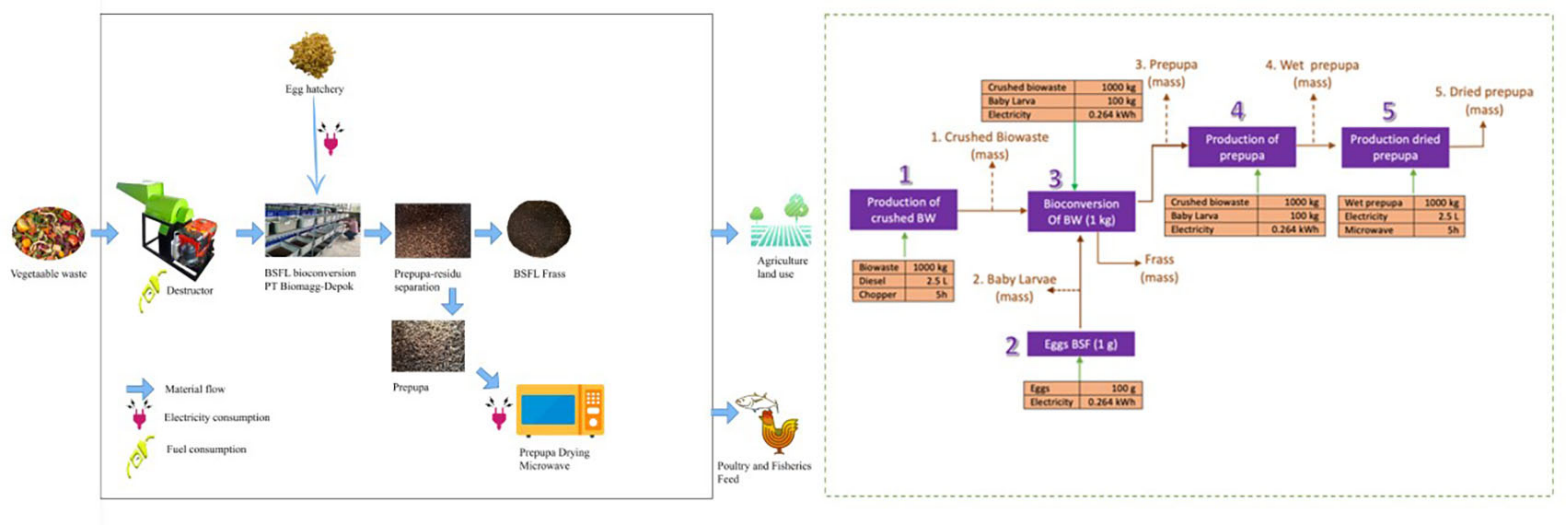
System boundary, process, and input flow of the BSFL farm for BSFL dried production at PT Biomagg Sinergi Internasional, Depok, West Java, Indonesia.

The current report is a preliminary study of the life cycle assessment and environmental impact analysis of BSFL farming in producing dried BSFL by using biowaste as a substrate for BSFL. The biowaste was provided from the traditional market, Depok, West Java, Indonesia. The study was located at PT Biomagg Sinergi Internasional, located in Depok, West Java, Indonesia (6°22′48.4″S 106°52′51.7″E). The system boundary (gate-to-gate) is designed for the core process of both processing and production of the dried larva. The present study used the functional unit as 100 kg of dried BSFL, which is an amount of dried BSFL production per cycle. Further, five environmental impact categories, GWP, AC, TE, FFE, and ET were chosen ( Figure 1-Left). The following processes were evaluated: 1) biowaste preparation for BSFL substrate, 2) egg hatching to produce baby larvae, 3) bioconversion of biowaste, 4) production of prepupa, and 5) production of the dried larva ( Figure 1-Right). Respectively, 1) at biowaste preparation for BSFL substrate, the volume of biowaste (1000 kg) and diesel for crushing biowaste and operation time of chopper machine were recorded. The biowaste was crushed using the chopper machine to homogenize the waste to make it easy to digest for BSFL. Meanwhile, 2) the number of eggs that were hatched (100 g), which were provided from PT Biomagg Sinergi Internasional, and the energy of electricity consumed (0.264 kWh) during the hatching process were noted. The eggs were incubated in the plastic box with crushed biowaste as substrate after they hatched. The egg was incubated for 3 days to produce baby larvae. 3) In the bioconversion of biowaste, the volume of biowaste (1000 kg), mass of baby larvae of BSFL (100 kg) and the energy of electricity consumed (0.264 kWh) were also recorded. Further, 4) the volume of crushed biowaste (1000 kg), baby larva (100 g), and electricity (0.264 kWh) during the production of prepupa were obtained and noted. Finally, 5) the wet prepupa (1000 kg), electricity energy consumed, and hour of microwave used in production of dried prepupa per 100 kg, were kept. All data in step 1–5 was used as life cycle inventory data for measuring impact assessment, as described below, and operation time of chopper machine were recorded.

The data used was starting from biowaste production, biowaste bioconversion, prepupa and frass production, and BSFL dried production. This data was primary data (volume of biowaste and diesel, the number of eggs, mass of baby larvae of BSFL and the energy of electricity consumed) that were directly taken from the PT Biomagg Sinergi Internasional ^19^ and was evaluated using the OpenLCA 1.11.0 (GreenDelta, Berlin), Ecoinvent database version 3.8 (Secondary data) and TRACI 2.1 method based on a gate-to-gate approach. Secondary data such as data biowaste, electricity, diesel, and chopper were obtained from the dataset of Ecoinvent 3.8 database. The Life Cycle Inventory (LCI) involved input waste (biowaste), emissions, and energy consumption of each subprocess, based on the principle of mass balance. The LCI involved input waste (biowaste), emissions, and energy consumption of each subprocess and were based on the principle of mass balance. Meanwhile, the impact environment that includes GWP, AC, TE, FFE, and, ET, were evaluated.

Additionally, all inventory data was obtained and calculated from this facility, except for CH _4_ and N _2_O emissions. The published values for CH _4_ and N _2_O emissions during BSFL bioconversion were used. ^20^ It was anticipated that residue during bioconversion produced emissions equivalent to ordinary organic waste from home or kitchen garbage. Furthermore, the results of the LCI evaluation may be utilized to examine life cycle impacts such as environmental implications. As previously stated, the relevant inventory resulted in the identification of five environmental impact categories. All methods have been deposited on protocols.io at: https://dx.doi.org/10.17504/protocols.io.8epv5j54dl1b/v1. ^21^

## Results and Discussion

The present report evaluated the LCA and environmental impact analysis of the dried BSFL production from biowaste bioconversion using BSFL in PT Biomagg Sinergi Internasional, Depok, West Java, Indonesia ( Table 1). ^19^

**Table 1.** Functional unit and value of the environmental impact analysis per 100 kg of the black soldier fly larvae (BSFL) dried production at PT Biomagg, Sinergi Internasional, Depok, West Java, Indonesia.

Impact category	Unit	Value
Global Warming Potential (GWP)	kg CO _2_-eq	6.687
Acidification (AC)	SO _2_-eq	0.029
Terrestrial eutrophication (TE)	kg N-eq	0.092
Fossil fuel depletion (FFD)	MJ surplus	14.767
Ecotoxicity (ET)	CTUe	119.264

**Figure 2.**
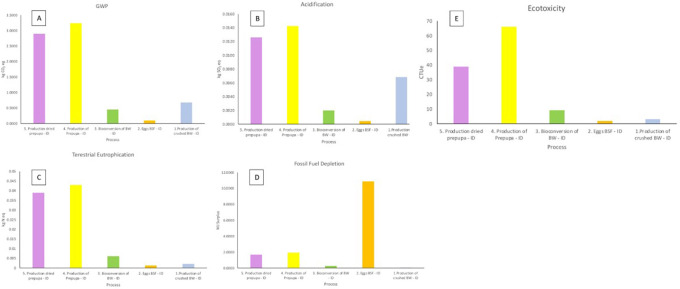
Impact analysis of the black soldier fly larvae (BSFL) dried production at PT Biomagg, Sinergi Internasional, Depok, West Java, Indonesia.

The GWP of the BSFL bioconversion system was calculated to be 6.687 kg CO2-eq. The specified amounts were 2.898 kg CO _2_-eq for dried BSLF production use, 3.239 kg CO _2_-eq for prepupa production, 0.452 CO _2_-eq for bioconversion of biowaste, 0.096 kg CO _2_-eq for eggs BSF hatching, and 0.680 kg CO _2_-eq for production crushed biowaste. A past study by Salomone *et al.,* ^22^ revealed that each 100 kg of food waste/biowaste emits 3.2 kg CO _2_ equivalent per global warming potential. Meanwhile, the greatest proportion (39.33%) of the overall energy usage was attributable to drying. Salomone, Saija ^22^ also stated that substantial GWP effects were generated by electricity use during the prepupa drying and using the microwave was related with the greatest energy consumption in the dried BSFL production system ( Figure 2A).

Meanwhile, acidification ( Figure 2B) was often associated with the pollutants which are resulted from N- compounds. The total effect of acidification was 0.029 kg SO _2_-eq. The present report stated that the emissions from the production of the prepupa process had the greatest influence on acidification. High NH _3_ emissions during the prepupa production caused a significant acidification burden. Further, the overall effect of NH _3_ emissions on terrestrial eutrophication was 0.092 kg N-equivalent. During the production of prepupa, emissions of NH _3_ accounted for most of the emissions, which was 0.0429 kg N-equivalent. In addition, the sum of the effect on fossil fuel depletion was 14.76 MJ surplus. The fossil fuel depletion produced by the production of crushed biowaste was 10.88 MJ surplus, which used a diesel-electric generating set in operating the chopper machine. Finally, the eco-toxicity for the system was 119.264 CTu. The eco-toxicity was related to electricity 66.017 CTu and 1.624 CTu in tap water used.

## Conclusion

This brief report revealed the GWP, the effects of acidification, terrestrial eutrophication, and eco-toxicity, bridging a significant information gap regarding the environmental impact of the BSFL bioconversion system. Contribution analysis might assist in locating “hot spots” within the selected environmental impact categories. Electricity and tap water for prepupa production, and electricity consumption for crushing biowaste, were the top three processes in terms of the GWP. This study also reported the environmental impact of the production of 100 kg of dried BSFL using the life cycle assessment method. Environmental impact analyzed includes the potential for global warming potential, acidification, terrestrial eutrophication, fossil fuel depletion, and eco-toxicity with their respective values of 6.687 kg CO _2_ eq; 0.029 SO _2_- eq; 0.092 kg N-eq; 14.767 MJ surplus; 119.264 CTUe. The prepupa production is the biggest contributor to global warming potential, acidification, terrestrial eutrophication, and eco-toxicity of all stages in dried BSFL production. It is suggested to use alternative single raw materials for substrate BSFL and another drying method, so that the sustainable BSFL dried production process can be achieved. Another recommendation is optimizing the use of tap water, by tightening the implementation of the SOP for tap water in order to be more economical and efficient for usage in BSFL dried production.

## Data Availability

Figshare: Life Cycle Assessment BSF, https://doi.org/10.6084/m9.figshare.22224034. ^19^ This project contains the following underlying data:
• Impact anaylisis assessment.xlsx (Present data shows raw data from the life cycle inventory to assess the impacts of BSFL dried production. The impacts assessment are: Global warming Potential, Acidification, Terestrial Eutrophication, Fossil Fuel Depletion, and Ecotoxicity) Data are available under the terms of the Creative Commons Zero “No rights reserved” data waiver (CC0 1.0 Public domain dedication). Protocol.io: Life Cycle Assessment for Black Soldier Fly Larvae Dried Production, https://dx.doi.org/10.17504/protocols.io.8epv5j54dl1b/v1. ^21^ Data are available under the terms of the Creative Commons Attribution 4.0 International license (CC-BY 4.0).
