# Supplementary material for: Bioconversion of biowaste by black soldier fly larvae ( Hermetia illucens L.) for dried larvae production: A life cycle assessment and environmental impact analysis
Source: F1000Res. 2023 Jul 11;12:814. [Version 1] doi: 10.12688/f1000research.132371.1 (PMC10477724; doi:10.12688/f1000research.132371.1)
Supplement: Peer review history

# Peer review: Reviewer response for version 1

**DOI:** 10.5256/f1000research.145285.r198614

**Published:** 2023-09-04

**Authors:** Pande Gde Sasmita Julyantoro

**Affiliations:** 1 Faculty of Marine Science and Fisheries, Universitas Udayana, Bali Province, Indonesia

This study reported a life cycle assessment (LCA) of the biowaste bioconversion process of BSFL and determined the environmental impact categories namely global warming potential (GWP), acidification (AC), terrestrial eutrophication (TE), fossil fuel depletion (FFE), eco-toxicity (ET). Interesting results showed that Electricity and tap water for prepupa production, and electricity consumption for crushing biowaste, were the top three processes in terms of the GWP and that prepupa production is the biggest contributor to global warming potential, acidification, terrestrial eutrophication, and eco-toxicity of all stages in dried BSFL production.

 The recommendation/suggestion of this study might be not only using alternative single raw materials for substrate BSFL, using another drying method, and optimizing the use of tap water but might also provide information on the optimum dried BSFL production when using a high-efficiency bioconversion process.

Is the work clearly and accurately presented and does it cite the current literature?

Yes

If applicable, is the statistical analysis and its interpretation appropriate?

Not applicable

Are all the source data underlying the results available to ensure full reproducibility?

Yes

Is the study design appropriate and is the work technically sound?

Yes

Are the conclusions drawn adequately supported by the results?

Yes

Are sufficient details of methods and analysis provided to allow replication by others?

Yes

Reviewer Expertise:

Aquatic microbiology; aquaculture

I confirm that I have read this submission and believe that I have an appropriate level of expertise to confirm that it is of an acceptable scientific standard.

# Peer review: Reviewer response for version 1

**DOI:** 10.5256/f1000research.145285.r186279

**Published:** 2023-07-18

**Authors:** Dino Rimantho

**Affiliations:** 1 Department of Industrial Engineering, Pancasila University, Jakarta, Special Capital Region of Jakarta, Indonesia

This study presents the bioconversion of biowaste by black soldier fly (Hermetia illucens L.) larvae for dry larvae production: Life cycle study and environmental impact analysis. This article uses LCA as a research methodology. This research uses open-source LCA, which is easy to apply. Furthermore, life cycle assessment (LCA) techniques are used and include (a) system boundary determination (gate-to-gate), starting with biowaste production, biowaste bioconversion, prepupae, and BSFL frass. This methodology adds to the literature on using BSFL Bioconversion through BSFL cultivation. It has the ability to decompose organic waste so that it can be an alternative solution in urban solid waste management. Some interesting points include:

 This article does not need to present statistical analysts because the method calculates life cycle analysis from Maggot BSF production. In addition, the authors have used LCA Open Source software as their analysis tools.

 In the introductory section, it is necessary to add BSFL application literature in other fields, such as health, renewable energy, animal feed, etc, so that the presence of this article can show the environmental impact of BSFL cultivation.

 In the results and discussion section, it is necessary to develop related to the weaknesses of this article so that it can become a recommendation for further research.

Is the work clearly and accurately presented and does it cite the current literature?

Yes

If applicable, is the statistical analysis and its interpretation appropriate?

Not applicable

Are all the source data underlying the results available to ensure full reproducibility?

Yes

Is the study design appropriate and is the work technically sound?

Yes

Are the conclusions drawn adequately supported by the results?

Yes

Are sufficient details of methods and analysis provided to allow replication by others?

Yes

Reviewer Expertise:

Environmental management, Waste Management, Environmental Engineering

I confirm that I have read this submission and believe that I have an appropriate level of expertise to confirm that it is of an acceptable scientific standard.
